# Bone bruising severity after anterior cruciate ligament rupture predicts elevation of chemokine MCP-1 associated with osteoarthritis

**DOI:** 10.1186/s40634-022-00478-8

**Published:** 2022-04-27

**Authors:** Lukas G. Keil, Douglas S. Onuscheck, Lincoln F. Pratson, Ganesh V. Kamath, Robert A. Creighton, Daniel B. Nissman, Brian G. Pietrosimone, Jeffrey T. Spang

**Affiliations:** 1 Department of Orthopaedic Surgery, School of Medicine, University of North Carolina, 130 Mason Farm Road, CB# 7055, Chapel Hill, NC 27599-7055 USA; 2 Department of Radiology, University of North Carolina, Chapel Hill, NC USA; 3 Department of Exercise and Sport Science, University of North Carolina, Chapel Hill, NC USA

**Keywords:** ACL injury, Bone bruising, Bone contusion, MCP-1, Posttraumatic osteoarthritis

## Abstract

**Purpose:**

Anterior cruciate ligament rupture is associated with characteristic bone contusions in approximately 80% of patients, and these have been correlated with higher pain scores. Bone bruising may indicate joint damage that increases inflammation and the likelihood of posttraumatic osteoarthritis. We sought to characterize the severity of bone bruising following acute anterior cruciate ligament injury and determine if it correlates with synovial fluid and serum levels of the proinflammatory chemokine monocyte chemoattractant protein-1 associated with posttraumatic osteoarthritis.

**Methods:**

This was a retrospective analysis of data collected prospectively from January 2014 through December 2016. All patients who sustained an acute ligament rupture were evaluated within 15 days of injury, obtained a magnetic resonance imaging study, and underwent bone-patellar-tendon-bone autograft reconstruction were offered enrollment. The overall severity of bone bruising on magnetic resonance imaging was graded (sum of 0–3 grades in 13 sectors of the articular surfaces). Serum and synovial fluid levels of monocyte chemoattractant protein-1 were measured within 14 days of injury, and serum levels were again measured 6 and 12 months following surgery. Separate univariate linear regression models were constructed to determine the association between monocyte chemoattractant protein-1 and bone bruising severity at each time point.

**Results:**

Forty-eight subjects were included in this study. They had a mean age of 21.4 years and were 48% female. Median overall bone bruising severity was 5 (range 0–14). Severity of bone bruising correlated with higher synovial fluid concentrations of monocyte chemoattractant protein-1 preoperatively (R^2^ = 0.18, *p* = 0.009) and with serum concentrations at 12 months post-reconstruction (R^2^ = 0.12, *p* = 0.04).

**Conclusions:**

The severity of bone bruising following anterior cruciate ligament rupture is associated with higher levels of the proinflammatory cytokine monocyte chemoattractant protein-1 in synovial fluid acutely post-injury and in serum 12-months following anterior cruciate ligament reconstruction. This suggests that severe bone bruising on magnetic resonance imaging after ligament rupture may indicate increased risk for persistent joint inflammation and posttraumatic osteoarthritis.

**Level of evidence:**

III ― retrospective cohort study.

## Background

Characteristic bone contusions occur in approximately 80% of patients who sustain an anterior cruciate ligament (ACL) injury [2, 11, 23, 27]. Bone bruising typically occurs when ligamentous insufficiency allows the posterolateral tibial plateau to abut the lateral femoral condyle as the mechanism of injury occurs [25]. The presence of bone bruising is associated with greater patient reported pain during the first four weeks post-injury as well as prolonged antalgic gait [15, 22]. However, bone bruising has not been shown to correlate with instability or functional outcomes [1, 3, 8, 12, 19]. Even in the absence of identifiable articular cartilage injury, subchondral bone bruising may indicate damage to the joint that increases acute inflammation and may hasten progression to posttraumatic osteoarthritis (PTOA) [7, 21]. PTOA prevention and treatment remain limited by an inability to identify patients at highest risk. Establishing an association between severe bone bruising, which is readily identified on magnetic resonance imaging (MRI), and biomarkers known to be associated with PTOA would allow for early identification of patients at increased risk for PTOA. This would facilitate further research and may eventually allow patient-specific delivery of treatments to minimize risk of PTOA following ACL rupture.

Separately, several synovial fluid biomarkers indicative of cartilage injury have been associated with development of arthritis, including the proinflammatory cytokine monocyte chemoattractant protein 1 (MCP-1), also known as chemokine C–C motif ligand 2 (CCL2) [6, 10, 13]. Elevated levels of MCP-1 have been correlated with radiographic and symptomatic PTOA after injury [4, 5, 9, 17, 20, 26, 28]. Bone bruising following ACL rupture has not yet been linked to increased levels of proinflammatory cytokines.

**Fig. 1:**
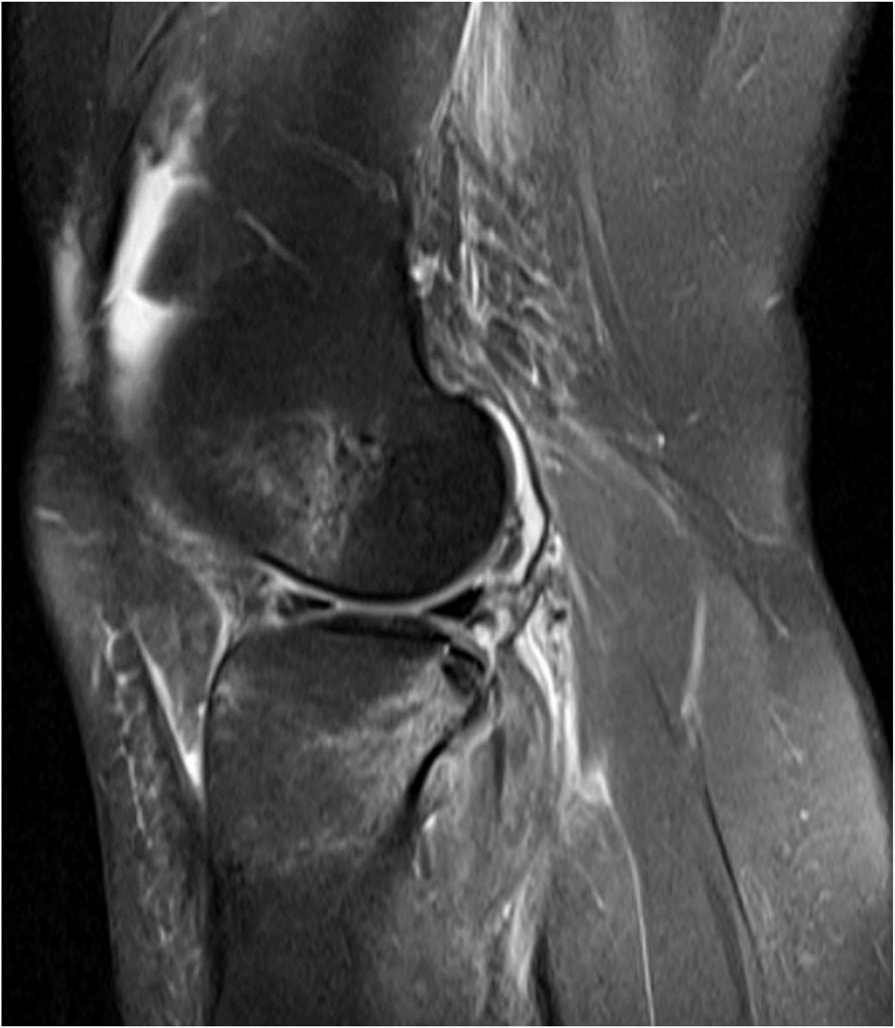
MRI post-ACL rupture demonstrating severe bruising in the lateral femoral condyle and posterior tibial plateau

The purpose of our study was to determine if the severity of bone bruising on MRI following acute ACL injury correlates with biomarkers associated with PTOA. We hypothesized that the severity of bruising as an indicator of damage to the joint would correlate with increased concentrations of the proinflammatory chemokine MCP-1 (CCL2) associated with posttraumatic osteoarthritis (Fig. 1).

## Methods

This was a retrospective analysis of prospectively collected data conducted as part of a study investigating clinical outcomes of ACL reconstruction (ACLR) at an academic medical center in the southeastern United States. It was approved by the local institutional review board. Informed consent was obtained from all subjects. Subjects were enrolled from January 2014 through December 2016. Enrollment was offered to all English-speaking patients aged 16 to 35 years with no prior diagnosis of inflammatory arthritis who sustained an acute ACL rupture, were evaluated within fifteen days of injury, obtained an MRI, and underwent bone-patellar-tendon-bone autograft ACLR.

### Analysis of bone bruising

MRI studies including T2 sequence images were obtained at the time of initial ACL injury diagnosis. MRIs were reviewed, and the severity of bone bruising was graded on T2 sequences in 13 sectors of the femoral and tibial articular surfaces using the scheme developed for the Whole-Organ Magnetic Resonance Imaging Score (WORMS) [24]. In this system the femoral and tibial articular surface are divided into six sectors each (the anterior, central, and posterior regions of the medial and lateral joint surfaces), and the region beneath the tibial spines is graded as a separate sector. In each sector the severity of bone bruising was graded 0–3, with 0 corresponding to no contusion, 1 to increased signal intensity in < 25% of the sector, 2 to 25–50%, and 3 to > 50% of the sector [24].

Each MRI was graded 4 times, twice by 2 resident physicians (orthopaedics and radiology) at least 6 weeks apart. Grades from all 4 reads were collated, and a final grade for each sector was assigned by a fellowship-trained musculoskeletal radiologist. The prevalence and severity of bone bruising in each sector was recorded. Reliability of this grading system was evaluated using kappa for intra- and inter-rater agreement [16]. The overall severity of bone bruising on MRI (sum of grades 0–3 in 13 sectors, theoretical range 0–39) was selected as the primary radiographic outcome, as logically it may reflect the relative energy of the mechanism of injury.

### Biomarker analysis

Serum and synovial fluid were collected via phlebotomy and sterile knee joint aspiration within 14 days of ACL injury, and serum was collected at 6 and 12 months following ACLR. Fluids were centrifuged and stored at -80 °C prior to batch analysis. Commercial enzyme-linked immunosorbent assays (ELISA) were used to evaluate concentrations of MCP-1 (ng/mL; R&D Systems, Minneapolis, MN, USA). The assay detection sensitivity for MCP-1 (mean minimal detectable dose) was reported as 1.7 pg/ml. All assays were performed in duplicate and exhibited < 10% inter- and intra-assay variability. These methods have been described in greater detail previously [18].

### Statistical analysis

Separate univariate linear regression models were constructed to determine the variance in MCP-1 levels associated with overall severity of bone bruising on MRI at each time point. *P*-values ≤ 0.05 were considered statistically significant. Data were analysed using SPSS version 16.0.0.2 [14].

## Results

Sixty-four patients were recruited for enrollment, of whom 14 were excluded due to history of prior ACLR. Two patients declined to have serum or synovial fluid collected for biomarker analysis and were also excluded. All 48 subjects who had an MRI obtained acutely post-injury and biomarker data at all time points were included. Subjects had a mean age of 21.4 years (SD 3.5) and were 48% female. Mean height was 175 cm (SD 11), and mean weight was 76 kg (SD 15).

### Bone bruising

On all 4 reads by both resident graders, there was bruising > 50% of the time in the lateral femoral central (LFc), lateral tibial posterior (LTp), and medial tibial posterior (MTp) regions, and there was bruising < 50% of the time in all other sectors (Table 1). Intra-rater agreement was best in LFc, LTp, and MTp sectors where bruising was common, with agreement ranging from 87–92% and kappa ranging from 0.54 (moderate) to 0.81 (near perfect). Intra-rater agreement in all other sectors where bruising was less common ranged from 90–100%, though Kappa (quickly affected by numerous zero values) was more variable, ranging from 0.27 (fair) to 1.0 (perfect). Combined inter-rater agreement in LFc, LTp, and MTp sectors was 80–90% with kappa ranging from 0.38 (fair) to 0.53 (moderate). Overall bone bruising severity (sum of grades in all sectors) ranged from 0 to 14 with median 5 and IQR 3.

**Table 1:** Prevalence of subarticular bone bruising by sector with intra- and inter-rater agreement

Sector^a^	Prevalence of Bone Bruising Among Final Grades	Intra-Rater Agreement^b^ (Agreement Kappa Interpretation)						Combined Inter-Rater Agreement^b^ (Kappa Interpretation)	
		Rater 1			Rater 2				
Lateral Femur Anterior	8%	95%	0.53	moderate	97%	0.00	slight		
**Lateral Femur Central**^a^	**84%**	**92%**	**0.81**	**near perfect**	**92%**	**0.77**	**substantial**	**0.52**	**moderate**
Lateral Femur Posterior	0%	98%	0.66	substantial	97%	0.00	slight		
Lateral Tibia Anterior	3%	98%	0.49	moderate	92%	0.27	fair		
Lateral Tibia Central	0%	97%	0.53	moderate	90%	-0.05	poor		
**Lateral Tibia Posterior**^a^	**93%**	**87%**	**0.59**	**moderate**	**88%**	**0.54**	**moderate**	**0.38**	**fair**
Tibial Spines	16%	91%	0.29	fair	90%	0.62	substantial		
Medial Femur Anterior	3%	100%	1.00	perfect	99%	0.79	substantial		
Medial Femur Central	13%	94%	0.65	substantial	95%	0.66	substantial		
Medial Femur Posterior	0%	―	―	N/A	98%	0.00	slight		
Medial Tibia Anterior	2%	98%	0.00	fair	100%	1.00	perfect		
Medial Tibia Central	0%	97%	0.48	moderate	98%	0.00	slight		
**Medial Tibia Posterior**^a^	**64%**	**90%**	**0.72**	**substantial**	**92%**	**0.72**	**substantial**	**0.53**	**moderate**

^a^Bolding indicates sectors where bruising was present on > 50% of all reads

^b^Weighted agreement (i.e., larger discrepancies between grades affect agreement more than smaller discrepancies, interpretations per Landis & Koch (1977)

### Correlation of bone bruising and biomarker data

The median concentration of MCP-1 in knee synovial fluid within 14 days of injury was 1261 ng/mL (IQR 1295), with higher concentrations observed among the top quartile of patients by severity of bone bruising on MRI and lower concentrations among the bottom quartile of patients by bone bruising severity on MRI. The trend towards higher MCP-1 concentrations among patients with more severe bone bruising was observed in serum concentrations also at all time points (Table 2). All MCP-1 concentrations exceeded the lower limit of detectability. Separate univariate linear regressions demonstrated that greater overall bone bruising severity correlated with higher synovial fluid concentrations of MCP-1 acutely post-injury (R^2^ = 0.18, *p* = 0.009) and with serum concentrations of MCP-1 at 12 months post-ACLR (R^2^ = 0.12, *p* = 0.04). There were no statistically significant associations between bone bruising severity and serum concentrations of MCP-1 acutely post-injury (R^2^ = 0.001; *P* = 0.799) or at 6 months post-ACLR (R^2^ = 0.07; *P* = 0.10).

**Table 2:** MCP-1 (monocyte chemoattractant protein 1) levels in knee synovial fluid and serum at three time points post-ACL rupture by severity of bone bruising on MRI. Values are median (IQR) in ng/mL

MCP-1 Concentration, median (IQR) in ng/mL	All Patients	Bottom Quartile of Bone Bruising (total bone bruising severity 0–2)	Top Quartile of Bone Bruising (total bone bruising severity 7–14)
Synovial fluid (< 2 weeks)	1261 (1295)	786 (1024)	1775 (1874)
Serum (< 2 weeks)	315 (126)	289 (62)	350 (161)
Serum (6 months)	294 (118)	270 (81)	305 (253)
Serum (12 months)	314 (166)	318 (137)	413 (288)

## Discussion

The findings of this study support our hypothesis that greater bone bruising severity post-ACL rupture is associated with elevated levels of the proinflammatory chemokine MCP-1. Notably, bone bruising was associated with higher MCP-1 levels both in synovial fluid preoperatively and in serum at 12-months post-injury. Bone bruising associated with ACL rupture has been studied extensively, but to date it has been correlated only with pain scores and not with instability or functional outcomes [1, 3, 8, 12, 15, 19, 22]. The current translational study provides a link between radiographic and biomarker data suggesting that greater bone bruising severity may be an indicator of increased risk of PTOA after ACL rupture.

The pattern and prevalence of bone bruising we observed is consistent with previous studies, with bruising occurring > 50% of the time in the lateral femoral condyle and posterior tibial plateau [7, 22]. We found that the severity of bone bruising on MRI can be reliably graded by orthopaedic surgeons and radiologists alike with good agreement between them. This suggests that clinicians could realistically note and quantify severe bone bruising in patients with acute ACL rupture and be able to better prognosticate their individual risks for persistent joint inflammation and PTOA.

This study has a number of limitations. It is non-randomized, but it assesses the natural history of ACL rupture rather than the effects of an intervention, making a prospective cohort study the ideal study design. Additionally, we offered enrollment consecutively to all eligible patients of all orthopaedic sports surgeons at our institution, minimizing selection bias. Retrospective studies are often susceptible to recall bias, but all data in the current study including biomarkers and MRI images were collected prospectively, mitigating this as a potential source of bias. Graded outcomes such as bone bruising severity are subject to experimenter’s bias (the tendency of values to drift towards the value expected by the rater), but the rating system we used with two raters making two passes each and a third rater to adjudicate discrepancies minimizes this as well. Finally, the distribution of bone bruising grades with many zero values is inherently non-normal, but the linear regressions used are tolerant of non-normal distributions, eliminating this as a source of error.

To our knowledge, this is the first study to demonstrate and association between higher synovial fluid concentrations of MCP-1 and bone bruising severity acutely post-ACL rupture. This suggests that MRI may be able to indirectly detect injuries likely to cause higher concentrations of pro-inflammatory cytokines, which could theoretically lead to early identification of those patients at greatest risk of developing post-traumatic osteoarthritis. Additionally, the severity of bone bruising at the time of ACL injury may be linked to higher serum concentrations of pro-inflammatory cytokines that persist 12 months post-ACLR. The lack of statistically significant correlations between bone bruising severity and serum concentrations of MCP-1 acutely and 6 months post-injury is of unclear significance, though the consistent trends observed in Table 2 at all time points suggest that a study with greater power might detect similar correlations at these time points as well. These data suggest that the severity of bone bruising at the time of ACL injury is linked to the inflammatory process acutely post-injury and 12 months post-ACLR that may be associated with the development of PTOA.

## Conclusions

The severity of bone bruising following ACL rupture is associated with higher levels of the proinflammatory chemokine MCP-1 (CCL2) in synovial fluid preoperatively and in serum 12-months post-ACLR. This suggests that severe bone bruising on MRI acutely post-ACL rupture may indicate greater joint inflammation, which may increase the risk for future posttraumatic osteoarthritis.

## Data Availability

The data used in this study can be provided upon reasonable request. They are not otherwise publicly available.

## Abbreviations

ACL: Anterior cruciate ligament
ACLR: Anterior cruciate ligament reconstruction
CCL2: Chemokine C–C motif ligand 2 (also known as MCP-1)
ELISA: Enzyme-linked immunosorbent assay
LFc: Lateral femoral central
LTp: Lateral tibial posterior
MCP-1: Monocyte chemoattractant protein 1 (also known as CCL2)
MRI: Magnetic resonance imaging
MTp: Medial tibial posterior
PTOA: Posttraumatic osteoarthritis
WORMS: Whole-Organ Magnetic Resonance Imaging Score
